# The Association between eHealth Capabilities and the Quality and Safety of Health Care in the Netherlands: Comparison of HIMSS Analytics EMRAM data with Elsevier’s ‘The Best Hospitals’ data

**DOI:** 10.1007/s10916-015-0274-7

**Published:** 2015-08-05

**Authors:** Rube van Poelgeest, Jan-Peter Heida, Lorren Pettit, Rob J. de Leeuw, Guus Schrijvers

**Affiliations:** Researcher at Julius Center, Public Health, UMC Utrecht, Utrecht, The Netherlands; Partner at SiRM - Strategies in Regulated Markets, The Hague, The Netherlands; Vice President Market Research HIMSS Analytics, Chicago, IL 60603/5616 USA; Psychologist/Senior Researcher at Julius Center, Public Health, UMC Utrecht, Utrecht, Netherlands; Health economist and former professor of public health at the University Medical Center Utrecht, Utrecht, Netherlands

**Keywords:** Hospital, Safety, Care, Quality, EMR

## Abstract

**Objective:**

To test the hypothesis that advanced electronic medical record (EMR) capabilities are associated with better quality and safety of hospital care.

**Methods and Findings:**

We used data from the HIMSS Analytics EMR Adoption Model (EMRAM^SM^) to measure the adoption and use of information technology in Dutch hospitals. To measure the quality and safety of healthcare in Dutch Hospitals we used select data from the publicly available basic set and the safety set of the Health Care Inspectorate (IGZ) and the Dutch Health Care Transparency Program ‘Zichtbare Zorg’ (ZIZO) program. The quality and safety measures selected reflect the measures used to score Dutch hospitals as presented in Elsevier’s annual ‘The Best Hospitals’ publication. The scores of this publication are based upon 542 of the 1516 available indicators from this basic set and safety set. Almost all indicators from the hospital-wide indicator sets are included in the selection, as are a large portion of indicators for acute care delivered by all hospitals. Of the 84 non-academic hospitals in the Netherlands, 67 (80 %) were included in this study.

**Results:**

There is no statistically significant association found between a hospital’s EMRAM score and their overall quality/safety performance in the Elsevier hospital scoring model.

**Conclusion:**

There is no evidence found to support the research hypothesis at this point in time. This outcome maybe the result of a multiplicity of factors to include the (limited) use of the methodologies used in this study, the fact that no fully digitalized hospital (EMRAM stage 7) is yet present in the NL, and/or the organizational competency of the NL hospitals in fully leveraging the EMR to facilitate patient care. Further research is needed to explore these findings.

## Introduction

Implementations of potentially transformative information technologies are currently underway internationally, often with significant impact on national expenditure [1, 2]. Such large-scale efforts and expenditures have been justified on the grounds that EMR, picture archiving and communication systems (PACS), electronic prescribing (ePrescribing) and associated computerized provider (or physician) order entry systems (CPOE), and computerized decision support systems (CDSS) are supposed to help to address the problems of variable quality and safety in modern health care [3] . However, the scientific basis of such claims, which are repeatedly made and seemingly uncritically accepted, remains to be firmly established [4–10]. This paper has the objective to contribute to the scientific discourse on the relationship between the digitalization of hospital care and quality and safety of such care by exploring the experience in one European country with fairly advanced EMR capabilities: The Netherlands. The hypothesis to be tested is: advanced electronic medical record (EMR) capabilities are positively associated with quality and safety of hospital care.

## Methods

For the measurement of the level of implementation of information systems the concept of maturity of information systems has been developed. There are a large number of methods or models available to measure the level of implementation of information technology [11]. This study will use the so-called Electronic Medical Record Adoption Model (EMRAM) scoring approach developed by Healthcare Information and Management Systems Society (HIMSS) Analytics [12]. EMRAM is an eight stage maturation model reflecting the EMR capabilities in hospitals, ranging from a completely paper-based environment (Stage 0) to a highly advanced digital patient record environment (Stage 7). The EMRAM model is perhaps one of the most commonly cited EMR maturation models in the world as it’s scoring approach has been applied to over 10.000 hospitals in the U.S., Canada, Europe, the Middle and Far-East and Australia. For a more detailed description of the HIMSS Analytics EMR Adoption Model, see [13].

To adjudicate a hospital’s EMR maturation, the CEO’s of every non-academic hospital in the Netherlands (84) were invited to participate in the EMRAM study. In the beginning of 2014, 67 hospitals (80 %) joined the program. The scoring process was done by identifying the software used in the different functional areas of the hospital. At least 150 questions per hospital were asked about demographics, software functionalities, processes, integration standards, usage in percentage by physician and nurses, depending on the available software in the hospital. In order to monitor the quality of the scoring process closely and distances in the Netherlands are never more than 200 km it was decided to do onsite visits. Depending on the complexity of the software environment, visits took between 1.5 and 4 h. For instance in the case of software from multiple vendors instead of one vendor identification of how the software is interconnected and integrated took more time. Validation was done by the quality assurance department of HIMSS Analytics Europe and the scoring was done by a proprietary scoring algorithm by HIMSS Analytics North America (Table 1). If a hospital received an EMRAM stage 6 score, an additional 59 questions were asked by a validation team of international peer inspectors mostly from stage 6 or 7 hospitals in the EU. Stage 6 hospitals can apply for a stage 7 validation, consisting of a 2 day visit of peer inspectors. One day will be used for presentations of predefined issues and one day for hospital visits to check life processes and the paperless status of the hospital. Until stage 5 the achieved score is secret to make participation to this study easy to decide. Two consecutive measurements with an interval of 18 months were taken. No stage 7 hospital was measured in the NL until to date (Dec 2014). One of the senior researchers of HIMSS Analytics is co-author of this study.

**Table 1:** Frequency distribution of EMRAM scores

EMRAM Score	Frequency	Percent	Cumulative Percent
7	0	0	0
6	7	10	10
5	32	48	58
4	2	3	61
2	25	37	99
1	1	1	100
0	0	0	100
Total	67	100	

**Fig. 1:**
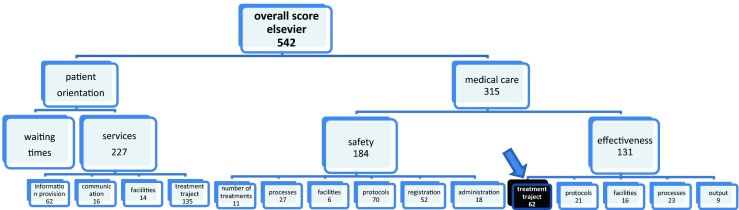
Structure of Elsevier scores based upon 542 indicators of publicity available hospital wide indicator sets

To measure the quality and safety of healthcare in Dutch Hospitals we used select data from the publicly available basic set and the safety set of the Health Care Inspectorate (IGZ) and the Dutch Health Care Transparency Program ‘Zichtbare Zorg’ (ZIZO) program (both sets survey year 2013). The quality and safety measures selected reflect the measures used to score Dutch hospitals as presented in Elsevier’s annual ‘The Best Hospitals’ publication. As the discussions about the transparency of the healthcare delivered in Dutch hospitals lasts, these are the best available data at this moment. Comparable reports are published, discussed and disputed in other countries [14–16]. The scores of Elsevier are, as opposed to other reports in the Netherlands, based upon publicly available indicators and based upon a scientific method to construct composite indicators [17]. This method has been prepared jointly by the OECD and the Applied Statistics and Econometrics Unit of the Joint Research Centre of the European Commission in Ispra, Italy. The scores of the Elsevier publication (Fig. 1) are based upon 542 of the 1516 publicly available indicators from the above mentioned datasets (IGZ and ZIZO).

Almost all indicators from the hospital-wide indicator sets are included in the above mentioned selection and a large portion of indicators for acute care. Some acute care is only delivered by specialized hospitals and cannot be used to compare all hospitals. Only those indicators for acute care are included that are delivered by all hospitals like infectious diseases, cardiovascular diseases and the surgical process. No indicators were selected for which a case mix correction is still necessary. In this study, the different indicators are combined into compound indicators. The annual reports have been collected and analyzed by ‘SiRM - Strategies in Regulated Markets’, a consultancy firm in The Hague. The indicators are first scaled to a uniform scale (z-score) and are then added together weighted. Hospitals that have not submitted data are given the scored lowest value. Hospitals could correct possible erroneous values: 68 hospitals have sent SIRM updates of their values. Care-related indicators are divided into the domains of ‘effectiveness’, ‘patient orientation’ and ‘safety’ (Fig. 1).

The scores in these three domains, together with waiting lists, determine the position of the hospital in the Elsevier study on ‘The Best Hospitals’. The scores on the domains of ‘safety’ and ‘effectiveness’ are bundled in a score for ‘medical care’. The scores on ‘waiting times’ and the domain ‘services’ in a score for ‘patient orientation’. The scores for ‘medical care’ and ‘patient orientation’ determine together the ‘total score’.

The score of a hospital is expressed in one to four balls (Table 2).

**Table 2:** Cross table of Elsevier scores and some underlying indicator sets

Elsevier score	overall		medical care		patient orientation		effective treatment	
	frequency	percent	frequency	percent	frequency	percent	frequency	percent
4	12	17,91	11	16,42	9	13,43	11	16,42
3	34	50,75	23	34,33	35	52,24	22	32,84
2	13	19,40	22	32,84	16	23,88	24	35,82
1	8	11,94	11	16,42	7	10,45	10	14,93
total hospitals	67	100,00	67	100,00	67	100,00	67	100

The balls do not contain any value judgment of Elsevier, but indicate how the hospital scores on the selected indicators compared with the average in the Netherlands. The participating hospitals do not qualify as “bad” or “good” in an absolute sense. The ‘effective treatment’ indicator (red box in Fig. 1) is part of the Elsevier effectiveness domain (Fig. 1) and is based upon 62 (only ZiZo) so called ‘structure’ indicators [18] per hospital. Elsevier and SiRM have made available the scores and all underlying data for the purpose of this study. One of the senior researchers of SiRM is co-author of this paper. Per hospital the 106 underlying EMRAM eHealth indicators and the 26 Elsevier indicators per hospital were included in a SPSS database. In a later stage also the mentioned 542 underlying basic indicators of the 26 Elsevier indicators were included to test the hypothesis of this paper.

## Results

No significant correlation is found between the EMRAM scores and the Elsevier performance indicators (Tables 3 and 4).

**Table 3:** Cross table of overall Elsevier scores and EMRAM scores

Overall Score Elsevier	EMRAM score								Total
	0	1	2	3	4	5	6	7	
4	0	0	3	0	1	6	2	0	12
3	0	1	14	0	1	14	4	0	34
2	0	0	3	0	0	9	1	0	13
1	0	0	5	0	0	3	0	0	8
Total	0	1	25	0	2	32	7	0	67

**Table 4:** correlation of EMRAM scores and Elsevier scores

Correlations		Overall Score Elsevier	Patient Orientation	Medical Care	Effectiveness	Effectiveness Treatment Traject
EMRAMscore	Pearson Correlation	,124	,081	,105	,075	-,223^*^
	Sig. (1-tailed)	,158	,258	,199	,272	,035
	N	67	67	67	67	67

*. Correlation is significant at the 0.05 level (1-tailed)

**Fig. 2:**
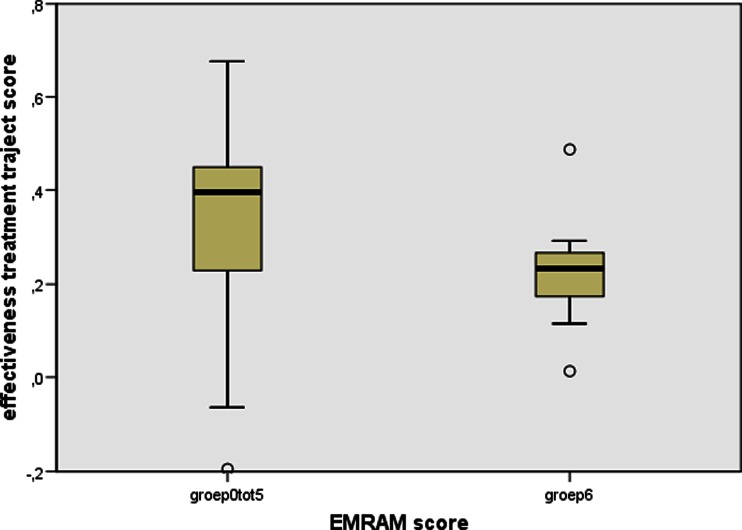
Comparison of median effective treatment score of EMRAM group 0–5 and group 6

Looking at underlying indicators, a one tailed significant (0.35 %) negative correlation (−0,223) (Fig. 2) is found between the EMRAM score and the Elsevier 2013 ‘effective treatment’ indicator (see red box in Fig. 1). This ‘effective treatment’ indicator is defined by Elsevier as ‘a measure for how the hospital organizes the treatment process for patients’. The box-plot of Fig. 2 also illustrates a negative correlation.

## Discussion

The hypothesis of this study, that there is a positive association between advanced electronic medical record (EMR) capabilities and quality and safety of hospital care in The Netherlands was not supported at this point in time by the findings of this study.

There are several reasons as to why these findings did not support the study hypothesis. For one, the models used to evaluate both the hospital’s EMR capabilities (the EMRAM model) and the quality and safety of hospital care in NL (the Elsevier model) may not be as sensitive as needed to capture the variances in performance outcomes. The EMRAM scoring approach for example, may over-inflate a hospital’s true EMR capabilities. While the EMRAM framework was designed to give guidance for the sequence of implementing EMR functionalities in hospitals by scoring hospitals on the “presence” of EMR tools, the “pervasiveness” of EMR tool use is not addressed until higher stages of the model. As such, hospitals could qualify as a stage 4 hospital if the required functionalities and facilities are implemented in only one patient care service area in the hospital even though other parts of the hospital reflect the capabilities of lower EMRAM stages. As such, it is possible that hospitals are not fully realizing the quality and safety benefits of their EMR because the tool’s use is not universally employed throughout the hospital, even though they are recognized as having fairly advanced EMR capabilities.

Secondly the Elsevier model. The scoring of the Elsevier model is mainly based (87 %) upon so called ‘structure’ indicators. The ‘effective treatment’ indicator is based upon 62 (100 %) ‘structure’ indicators. ‘Outcome’ or ‘process’ indicators are generally considered as better indicators for quality of care [18]. Transparency of hospitals is a big issue in the NL (as is abroad) because even if outcome indicators are measured they are most of the time not available for publication. To illustrate the dispute in the Netherlands, the ministry of Health made 2015 the year of the transparency. However, it could be that not only methodological limitations in this paper explain the absence of a positive relation between digitalization and quality of care. Recent literature [19, 20] has indicated and discussed comparable findings.

In the study of Jarvis [16] of 2988 hospitals with EMRAM scores in the USA, 248 were classified as ‘advanced EMR use’ (EMRAM stage 6 or 7). The remaining hospitals were classified as ‘non-advanced EMR use’. Estimated clinical process of care and patient experience of care scores were calculated by the American Hospital Association (AHA) by using data from Hospital Compare. Before adjusting for hospital characteristics (#beds, system status, teaching hospital, profit, and geographic region) EMRAM stage 7 users had significant higher clinical process scores and significant lower experience of care scores. After controlling for hospital characteristics, EMRAM stage 7 advanced EMR use was associated with significantly higher process of care scored than both EMRAM stage 6 advanced users and non-advanced users. There was no difference in process of care scores between EMRAM stage 6 advanced use and non-advanced use. After adjusting for hospital characteristics, there was no difference in experience of care scores by level of advanced use. These findings may support our conclusion that EMRAM stage 6 may not be a good enough indicator for advanced EMR use, because hospitals could qualify as a stage 6 hospital if the required functionalities and facilities are implemented in only one patient care service area in the hospital even though other parts of the hospital reflect the capabilities of lower EMRAM stages. Only at stage 7 the required functionalities and facilities are implemented in every patient care service area in the hospital. The number of hospitals in the NL (67) may not be enough to adjust for hospital characteristics in our study. No significant difference between hospital characteristics and EMRAM score was found in our study (Data available at the first author).

## Conclusion

The hypothesis of this study, that there is a positive association between advanced electronic medical record (EMR) capabilities and quality and safety of hospital care in the Netherlands, was not supported by the findings of this study at this point in time. This outcome may be caused by a multiplicity of factors (such as the characteristics of the models being used, the varied EMR implementation strategies employed by hospital leaders in the Netherlands, and/or the mastery of the staff in using these technologies) leading one to conclude that future research efforts should give careful consideration to these variables.
